# Photobiomodulation Therapy Decreases Oxidative Stress in the Lung Tissue after Formaldehyde Exposure: Role of Oxidant/Antioxidant Enzymes

**DOI:** 10.1155/2016/9303126

**Published:** 2016-05-17

**Authors:** Rodrigo Silva Macedo, Mayara Peres Leal, Tarcio Teodoro Braga, Éric Diego Barioni, Stephanie de Oliveira Duro, Anna Carolina Ratto Tempestini Horliana, Niels Olsen Saraiva Câmara, Tânia Marcourakis, Sandra Helena Poliselli Farsky, Adriana Lino-dos-Santos-Franco

**Affiliations:** ^1^Postgraduate Program in Biophotonics Applied to Health Sciences, University Nove de Julho (UNINOVE), Rua Vergueiro, 235/249 Liberdade, 01504-001 São Paulo, SP, Brazil; ^2^Department of Immunology, University of São Paulo, São Paulo, Brazil; ^3^Department of Clinical and Toxicological Analyses, Faculty of Pharmaceutical Sciences, University of São Paulo, São Paulo, Brazil

## Abstract

Formaldehyde is ubiquitous pollutant that induces oxidative stress in the lung. Several lung diseases have been associated with oxidative stress and their control is necessary. Photobiomodulation therapy (PBMT) has been highlighted as a promissory treatment, but its mechanisms need to be better investigated. Our objective was to evaluate the effects of PBMT on the oxidative stress generated by FA exposure. Male Wistar rats were submitted to FA exposure of 1% or vehicle (3 days) and treated or not with PBMT (1 and 5 h after each FA exposure). Rats treated only with laser were used as control. Twenty-four hours after the last FA exposure, we analyzed the effects of PBMT on the generation of nitrites and hydrogen peroxide, oxidative burst, glutathione reductase, peroxidase, S-transferase enzyme activities, the gene expression of nitric oxide, cyclooxygenase, superoxide dismutase, the catalase enzyme, and heme oxygenase-1. PBMT reduced the generation of nitrites and hydrogen peroxide and increased oxidative burst in the lung cells. A decreased level of oxidant enzymes was observed which were concomitantly related to an increased level of antioxidants. This study provides new information about the antioxidant mechanisms of PBMT in the lung and might constitute an important tool for lung disease treatment.

## 1. Introduction

Oxidative stress is characterized by the imbalance between an increased generation of reactive oxygen and nitrogen species (RONS) and a reduced antioxidant capacity. RONS are produced in high quantities by endogenous metabolisms which are represented primarily by the activation of neutrophils, the products of cyclooxygenase (COX), lipoxygenase (LOX), and nitric oxide synthases (NOS) [1–3]. At the same time, RONS are also produced in the body through exogenous factors including formaldehyde (FA), particulate matter (PM), metals, and quinones [4–9].

We have identified oxidative stress as an important pathway in which FA exerts its toxic effects, with a disruption of the lung physiological balance between the oxidant and antioxidant systems, modulating positively with a neutrophilic lung inflammation [9, 10]. FA is ubiquitous pollutant found in many industries, offices, and laboratories, and it is also emitted in the domestic ambient of homes, in things such as furniture, building materials, and chipboards, and in heating and cooking fumes [11].

Neutrophils that are activated by different biochemical mechanisms produce high quantities of RONS and inflammatory cytokines, leading to a severe destruction of the lung tissues [12, 13]. However, the deleterious effects of RONS are neutralized naturally by the lung through the defensive antioxidant system. Among the main antioxidant sources in the lung are glutathione reductase, peroxidase, s-transferase enzymes, superoxide dismutase, the catalase enzyme, and heme oxygenase-1 [14–16].

Several lung diseases such as asthma, chronic obstructive pulmonary diseases, and lung fibrosis have been associated with oxidative stress [17–19]. Lung diseases constitute an important public health problem and the control of oxidative stress into the lung is necessary. In this context, photobiomodulation has been highlighted as a promissory treatment, because of the absence of side effects, displaying low costs, and a noninvasiveness. However, its mechanisms need to be better investigated and understood.

Some studies have evaluated the antioxidant effects of laser therapy by using different models in vivo and in vitro [20–23]. When considering the antioxidant effects of PBMT in the lung tissues, studies have reported that, in an animal experimental model, laser treatment restored the balance between the oxidant and the antioxidant mediators, raising the PPAR expression and consequently the production of HSP70 [22].

Based upon the oxidative stress that is induced by FA exposure in the lung tissues and the antioxidant effects of PBMT, we evaluated the generation of nitrites and hydrogen peroxide, oxidative burst, glutathione reductase, peroxidase, s-transferase enzyme activities, the gene expression of nitric oxide, cyclooxygenase, superoxide dismutase, the catalase enzyme, and heme oxygenase-1. Thus, this study may provide information about the antioxidant mechanisms of PBMT in lung diseases.

## 2. Materials and Methods

The experiments were approved by the Committee on the Ethics of Animal Experiments of the University of Nove de Julho (CoEP-UNINOVE, Permit Number: AN0029.2014).

### 2.1. Animals

Male 2-month-old Wistar rats (40) were obtained from the University Nove de Julho and maintained in a light and temperature-controlled room (12/12-hour light-dark cycle, 21 ± 2°C), with free access to food and water.

### 2.2. Formaldehyde (FA) Exposure

Group of rats (5/chamber) were exposed to FA inhalation (1%, 90 min/day) or vehicle (distilled water) for 3 consecutive days. Thus, we utilized a standard glass chamber (20 L) coupled to an ultrasonic nebuliser device (Icel®, Brazil) which produces an aerosol with particles between 0.5 and 1 micron to generate a constant airstream in an aqueous solution of formalin [9, 10, 24].

### 2.3. Photobiomodulation Therapy

According to Miranda da Silva et al. [25], rats received infrared laser (CW Diode Laser-MMOptics, São Paulo, Brazil) 1 and 5 h after each FA or vehicle inhalation. The irradiation was performed directly in the skin in nine points of respiratory tract (3 points in the trachea and 3 points in the right and left lung lobes). After 24 h of last FA exposure the analyses were performed. We used the following parameters: output power of 30 mW, 660 nm wavelength, 60 s/point, and spot size of 0.14 cm^2^, resulting in an irradiance of 210 mW/cm^2^ and radiant exposure of 12.86 J/cm^2^. The optical power was calibrated using a Newport 1835 C multifunction optical power meter (Equipland, Oklahoma Road, Sao Jose, CA, USA). The laser power was monitored during laser irradiation by collecting laser light with a partial reflection (4%) mirror. The laser irradiation dose was set at 1.8 J for 1 min [24–28].

### 2.4. Experimental Groups

The rats were assigned into 4 experimental groups: N, nonmanipulated rats; FA, identified as rats submitted to FA inhalation; L, identified as rats treated only with laser; and FA + L, identified as rats subjected to FA inhalation and treated with laser. The rats were killed by sectioning the abdominal aorta under deep anaesthesia with ketamine-xylazine by intraperitoneal route (100 mg/kg and 20 mg/kg, resp.) 24 h after the last FA inhalation.

### 2.5. Quantification of Nitrites in the Supernatant of Bronchoalveolar Lavage (BAL)

The concentration of nitrites (NO_2_) was determined in the BAL supernatants samples. Nitrites levels were quantified according to the Griess method. The optical density (540 nm) was recorded using a microplate reader (Bio-Tek Instr., USA) and the nitrites levels were obtained using a standard curve of NaNO_2_ (5–60 *μ*M).

### 2.6. Quantification of Hydrogen Peroxide in the Bronchoalveolar Lavage (BAL) Cell Suspension

The hydrogen peroxide levels were quantified in sample of BAL cells. The BAL cell suspension (1 × 10^5^ cells/mL in phenol red solution) was stimulate with PMA (10 ng/well) and incubated at 37°C, 5% CO_2_ for 1 hour. After this time, the reaction was stopped by the addition of 10 *μ*L of NaOH 1 N. The optical density (620 nm) was recorded using a microplate reader (Bio-Tek Instr., USA) and the hydrogen peroxide levels were obtained using a standard curve of H_2_O_2_ (0–200 nM) and expressed in % H_2_O_2_/1 × 10^4^ cells.

### 2.7. Determination of Gene Expression of Oxidants and Antioxidants Enzymes in the Lung Tissue

Lung samples were snap-frozen in liquid nitrogen. The total RNA was isolated from lung tissue using Trizol Reagent (Invitrogen, Carlsbad, CA) according to Invitrogen. RNA concentrations were determined by spectrophotometer absorbance readings at 260 nm. First-strand cDNAs were synthesized using the MML-V reverse transcriptase (Promega, Madison, WI). RT-PCR was performed using the SYBR Green real-time PCR assay (Applied Biosystem, USA) for the following molecules: hypoxanthine guanine phosphoribosyl transferase (HPRT) (sense) 5′-CTC ATG GAC TGA TTA TGG ACA GGA C-3′ and (antisense) 5′-GCA GGT CAG CAA AGA ACT TAT AGC C-3′; iNOS (sense) 5′-AGT GAG GAG CAG GTT GAG GA-3′ and (antisense) 5′-GCT GTA ACT CTT CTG GGT GT-3′. RT-PCR was performed using the TaqMan real-time PCR assay (Applied Biosystem, USA) for the following molecules: COX-1 (Rn00566881_m1^*∗*^), COX-2 (Rn01483828_m1^*∗*^), SOD-1 (Rn00566938_m1^*∗*^), SOD-2 (Rn00690587_g1^*∗*^), and catalase (Rn00560930_m1^*∗*^). Cycling conditions were as follows: 10 min at 95°C followed by 45 cycles at 20 s each at 95°C, 20 s at 58°C, and 20 s at 72°C. Analysis was performed using Sequence Detection Software 1.9 (SDS), and mRNA expression was normalized to HPRT expression.

### 2.8. Quantification of Enzymatic Activities of Glutathione Peroxidase, Reductase, and S-Transferase

Glutathione peroxidase (GPx) activity was determined using* tert*-butylhydroperoxide as the substrate, and the formation of oxidized glutathione (GSSG) was indirectly monitored through NAPDH consumption (5 min, wavelength of 340 nm). Glutathione reductase (GR) activity was determined by the reduction of GSSG to GSH measured through NADPH consumption, which was monitored spectrophotometrically (10 min, wavelength of 340 nm, 37°C). Glutathione S-transferase (GST) activity was determined by measuring the conjugation of 1-chloro-2,4-dinitrobenzene (CDNB) with reduced glutathione. The formation of the complex was monitored spectrophotometrically (5 min, wavelength of 340 nm, 25°C). The GPx, GR, and GST assays were performed in a Power Wave ×340 spectrophotometer (Bio-Tek Instruments INC, software KC4 v3.0).

### 2.9. Evaluation of Oxidative Burst in the Bronchoalveolar Lavage (BAL) Cells

Quantification of oxidative burst was performed using BAL total cells (2 × 10^5^ cells/well) estimated by means of 2′,7′-dichlorofluorescin diacetate (DCFH-DA) fluorescence cells. Direct measurement of the mean fluorescence recorded on green channel was recorded as oxidative burst. A flow cytometer (FACS Calibur, Becton Dickinson Immunocytometry Systems, San Jose, CA, USA) interfaced with a Macintosh G4 computer was used. Data from 10,000 events were collected in list mode and analyzed in Cell Quest (Becton Dickinson Immunocytometry Systems). Fluorescence data were plotted on log scale. Green fluorescence from DCFH was measured at 530 ± 30 nm (FL1 detector).

### 2.10. Statistical Analysis

The statistical analysis was performed using the GraphPad Prism software (GraphPad Software, Inc.). The normality test was performed using Kolmogorov-Smirnov test. Since the data were parametric, we used one-way ANOVA followed by Student's Newman-Keuls. Differences were considered significant when *P* < 0.05.

## 3. Results

### 3.1. Photobiomodulation Therapy Reduced the Generation of Hydrogen Peroxide (H_2_O_2_) and Nitrites (NO_2_) Induced by FA Exposure in the Lung

**Figure 1:**
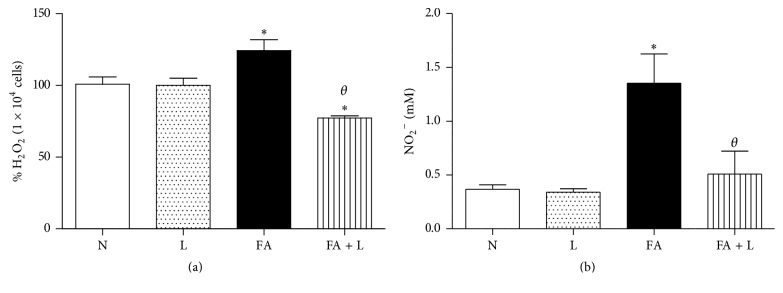
Photobiomodulation therapy reduces generation of H_2_O_2_ and NO_2_ after FA exposure in the lung. Group of rats was exposed or not to FA inhalation (1%, 90 min/day, 3 days) and treated or not with laser (30 mW, 1.8 J, 60 s/point, total 540 s, 1 and 5 h after each FA inhalation). In parallel, group of rats were treated only with laser and nonmanipulated rats were used to obtain basal parameters. The quantification of H_2_O_2_ and NO_2_ (a, b) was determined 24 h after the last FA inhalation. Data mean ± SEM of 6 animals per group. ^*∗*^ *P* < 0.05 in relation to N and L groups; ^*θ*^ *P* < 0.05 in relation to FA group.

To investigate the effects of PBMT on generation of RONS in the lung tissue, we quantified the H_2_O_2_ and NO_2_ in the bronchoalveolar lavage. Data of Figure 1 showed that treatment with laser reduced the levels of H_2_O_2_ (a) as well as NO_2_ (b) when compared to nontreated group (FA group) and did not differ from nonmanipulated and laser groups (N and L groups). We can also observe that FA exposure increased the generation of NO_2_ and H_2_O_2_ in relation to basal N and L groups.

### 3.2. Photobiomodulation Therapy Increased the Oxidative Burst in the Lung

**Figure 2:**
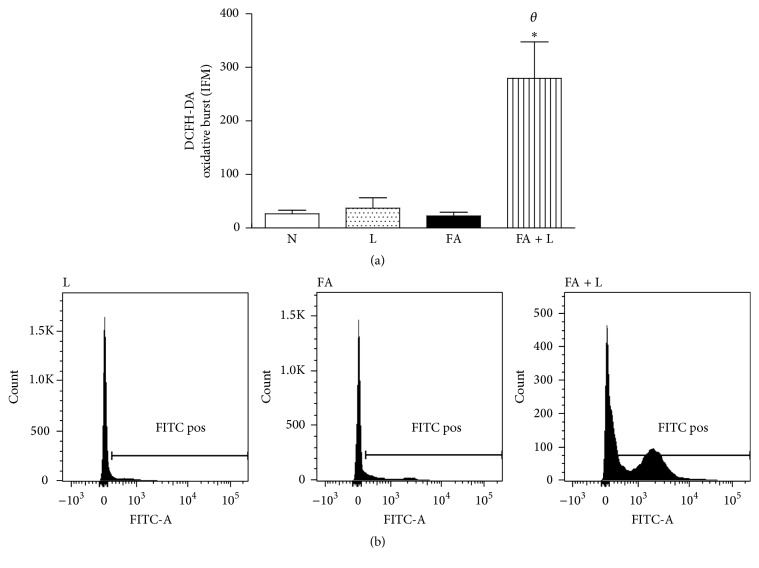
Photobiomodulation therapy increases oxidative burst in the lung. Group of rats was exposed to FA inhalation (1%, 90 min/day, 3 days) and treated or not with LLLT (30 mW, 1.8 J, 60 s/point, total 540 s, 1 and 5 h after each FA inhalation). In parallel, group of rats were treated only with laser and nonmanipulated rats were used to obtain basal parameters. The oxidative burst was determined 24 h after the last FA inhalation. Data mean ± SEM of 6 animals per group. ^*∗*^ *P* < 0.05 in relation to N and L groups; ^*θ*^ *P* < 0.05 in relation to FA group.

In order to investigate the effects of PBMT on the functional state of BAL cells, we evaluated the oxidative burst. Figures 2(a) and 2(b) showed that the treatment with laser increased the oxidative burst in BAL cells when compared to the FA, N, and L groups.

### 3.3. Photobiomodulation Therapy Reduced the Gene Expression of Nitric Oxide Synthase (iNOS and cNOS) and Cyclooxygenase (COX-2) Induced by FA Exposure in the Lung

**Figure 3:**
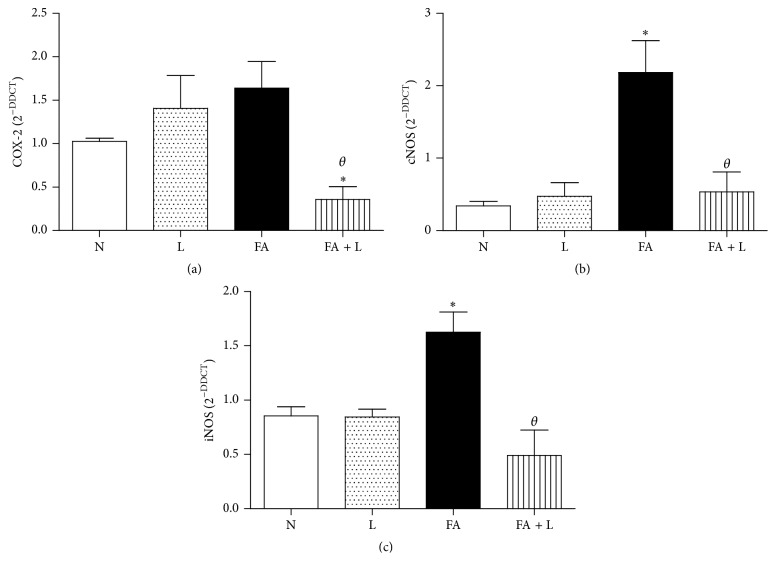
Photobiomodulation therapy decreases oxidant enzymes after FA exposure in the lung. Group of rats was exposed to FA inhalation (1%, 90 min/day, 3 days) and treated or not with LLLT (30 mW, 1.8 J, 60 s/point, total 540 s, 1 and 5 h after each FA inhalation). In parallel, group of rats were treated only with laser and nonmanipulated rats were used to obtain basal parameters. The oxidant enzymes were determined 24 h after the last FA inhalation. Data mean ± SEM of 6 animals per group. ^*∗*^ *P* < 0.05 in relation to N and L groups; ^*θ*^ *P* < 0.05 in relation to FA group.

In order to understand the possible mechanism involved in reactive oxygen and nitrogen species (RONS) after PBMT, we investigated important enzymes that generate RONS.  Figure 3(a) showed that PBMT reduced the gene expression of COX-2 when compared to the N, L, and FA groups. No differences were observed between FA, L, and N groups.

In Figures 3(b) and 3(c), we can observe that PBMT decreased the gene expression of cNOS and iNOS, respectively, when compared to the FA group and did not differ from L and N groups. We also showed that FA exposure increased the gene expression of both enzymes (cNOS and iNOS) in relation to nonmanipulated rats (N group) and rats treated only with laser (L group).

### 3.4. Photobiomodulation Therapy Increased the Gene Expression of Superoxide Dismutase (SOD-1 and SOD-2) and Heme Oxygenase-1 (HO-1) without Changing Catalase after FA Exposure in the Lung

**Figure 4:**
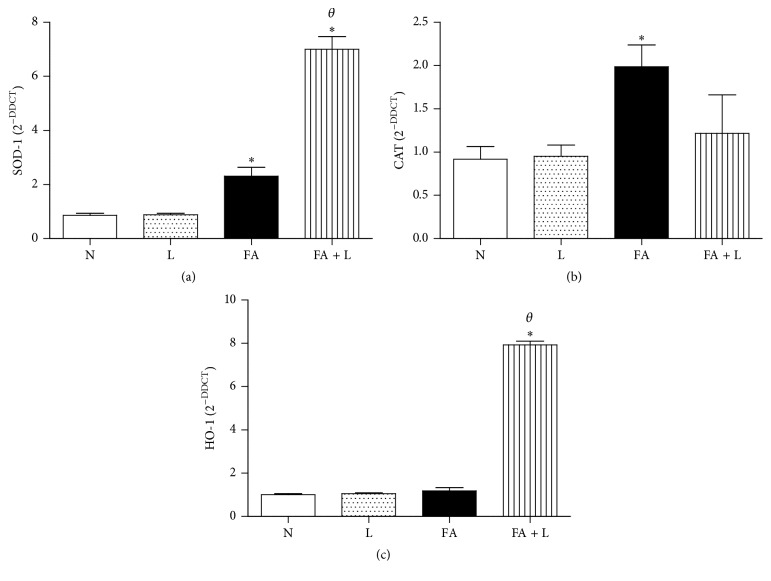
Photobiomodulation therapy increases antioxidant enzymes after FA exposure in the lung. Group of rats was exposed to FA inhalation (1%, 90 min/day, 3 days) and treated or not with LLLT (30 mW, 1.8 J, 60 s/point, total 540 s, 1 and 5 h after each FA inhalation). In parallel, group of rats were treated only with laser and nonmanipulated rats were used to obtain basal parameters. The antioxidant enzymes were determined 24 h after the last FA inhalation. Data mean ± SEM of 6 animals per group. ^*∗*^ *P* < 0.05 in relation to N and L groups; ^*θ*^ *P* < 0.05 in relation to FA group.

We investigated the involvement of PBMT in the gene expression of antioxidant enzymes.  Figure 4(a) showed that PBMT increased the gene expression of SOD-1 when compared to the FA, L, and N groups. On the other hand, in Figure 4(b) we showed that no differences were observed in the gene expression of catalase when rats were treated with laser (FA + L group). We also showed that FA exposure increased the gene expression of catalase in relation to control groups (N and L).

In Figure 4(c) we can observe that PBMT increased the gene expression of HO-1 when compared to the FA, L, and N groups. We also observed that FA exposure did not induce alteration in the expression of HO-1 in relation to control groups (N and L).

### 3.5. Photobiomodulation Therapy Increased the Activity of Glutathione S-Transferase (GST) and Peroxidase (GPX) without Changing Glutathione Reductase (GR) after FA Exposure in the Lung

**Figure 5:**
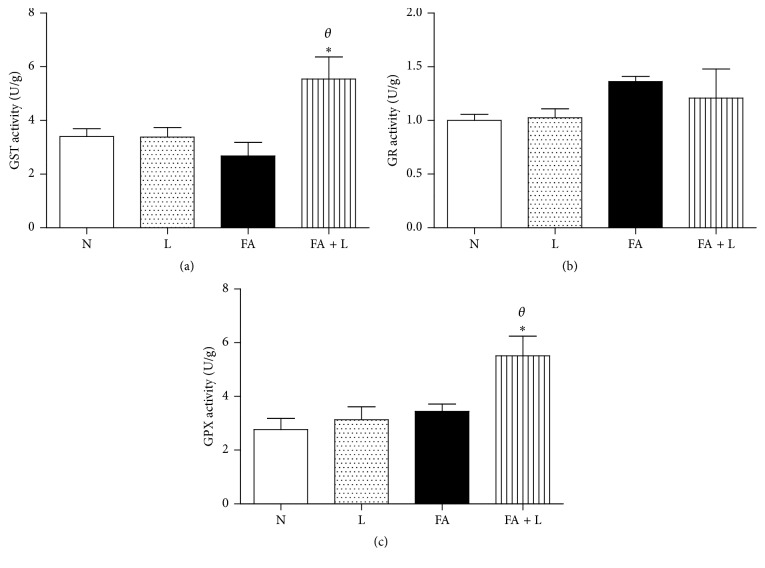
Photobiomodulation therapy increases activity of glutathione s-transferase and peroxidase enzymes after FA exposure in the lung. Group of rats was exposed to FA inhalation (1%, 90 min/day, 3 days) and treated or not with LLLT (30 mW, 1.8 J, 60 s/point, total 540 s, 1 and 5 h after each FA inhalation). In parallel, group of rats were treated only with laser and nonmanipulated rats were used to obtain basal parameters. The activities of glutathione s-transferase, reductase, and peroxidase were determined 24 h after the last FA inhalation. Data mean ± SEM of 6 animals per group. ^*∗*^ *P* < 0.05 in relation to N, and L groups; ^*θ*^ *P* < 0.05 in relation to FA group.

We also investigated the involvement of PBMT in the activity of glutathione enzymes that exert an important protection of oxidant species in the lung tissue. Figures 5(a) and 5(c) showed that PBMT increased the activity of GST and GPX, respectively, when compared to the FA, L, and N groups. On the other hand, PBMT did not interfere in the GR activity (Figure 5(b)).

## 4. Discussion

Photobiomodulation therapy was an effective treatment for oxidative stress that was induced by an FA exposure in the lung tissue, as it reduced the generation of H_2_O_2_, NO_2_, and the gene expression of oxidant enzymes that were concomitantly related to the increased gene expression of antioxidant enzymes. Photobiomodulation therapy also increased the activity of glutathione enzymes (GST and GPX) which are highly important for the protection of the lung against oxygen and nitrogen reactive species (RONS).

When considering the fact that oxidative stress is an important pathway by which FA induces a lung inflammation, we have shown here the protective effects of PBMT. As we expected, FA exposure evoked an increased generation of RONS in the cells recruited into the lung with respect to H_2_O_2_ and NO_2_, which was reversed by PBMT. These results can explain the previous results published by our group that showed a reduced lung inflammation after PBMT [25]. This is since RONS has been implicated in initiating inflammatory responses in the lungs, through the activation of transcription factors, such as the nuclear factor NF-*κ*B, leading to an enhanced gene expression of proinflammatory mediators.

The beneficial effects of PBMT were corroborated by a determination of the oxidative burst in the BAL cells that were predominantly neutrophils. The oxidative burst exerts an important role in the killing activity by the generation of oxygen reactive species by neutrophils. An elevated oxidative burst was found after PBMT, showing that this treatment improves the capacity of neutrophils to defend the body against microorganisms, although this process generates reactive species. Thus, we must consider that reactive species exert a dual role in the organism, protecting and/or prejudicing.

PBMT reduced the generation of NO_2_ and H_2_O_2_ and simultaneously increased the oxidative burst. These data could be considered controversial if it is not taking into account that several pathways on phagocytes can generate reactive species, which are simultaneously inactivated by antioxidant enzymes. In this context, phagocytes, such as macrophages and neutrophils, generate reactive species via oxidative burst, regulated by NADPH-oxidase. This latter enzyme generates superoxide radical that is subsequently converted into hypochlorous acid, a potent bactericidal agent. Thus, increased oxidative burst may be observed in reduced levels of H_2_O_2_. In addition, different mechanisms of defense against H_2_O_2_ production are available, such as glutathione peroxidase that was increased by PBMT, which contribute to reduced levels of H_2_O_2_. It is important to mention that our results corroborate those obtained by Dolgushin et al. [29], which showed that PBMT increased killing activity by neutrophils.

Since, in previous studies, we have shown that FA exposure causes a disruption of the physiological balance between the oxidant and antioxidant enzymes in the lung tissue, most likely by favoring the oxidant pathways and thus positively modulating the lung's inflammation [11, 20], we have investigated the effects of PBMT on the gene expression of these oxidant and antioxidant enzymes in the lung tissue.

Based on previous studies, we suppose that RONS produced during FA inhalation, generated by oxidant/antioxidant enzymes imbalance in the lung tissue, might alter the metabolism of lung phagocytes, which in turn could increase the release of inflammatory mediators, as well as reactive species, amplifying the lung inflammatory response [30]. In addition, it is reasonable to admit that we analyzed enzymes in the whole lung tissue, including parenchyma, muscle cells, structural cells, and phagocytes.

Our data has shown that PBMT reversed the increased gene expression of cNOS and iNOS in the lung tissue after FA exposure. These results corroborate with the reduced NO_2_ released by the BAL cells. Similarly, PBMT also reduced the gene expression of COX-2 that is an important oxidant enzyme and one that generates potent inflammatory mediators including eicosanoids. We can infer this reduction in the COX, as well as in the NOS, and where this is caused by a PBMT, these results might be responsible, at least in part, for the decreased generation of oxidative species released during an FA exposure and culminating in a reduced lung inflammation, as noted previously [25].

In the literature few studies showed the PBMT effects in model of lung diseases [22, 25–28]. Almost the works showed the anti-inflammatory and antioxidants effects of PBMT in experimental models of arthritis. Thus, studies that investigate the effects of PBMT in lung diseases are important, showing the alternative therapy without side effects.

As mentioned above, we also evaluated the antioxidant enzymes including SOD, CAT, HO-1, GPX, GR, and GST. The increased gene expression of SOD and HO-1 demonstrated the protective antioxidant mechanisms that are induced by PBMT. Thus, we can infer that the SOD and HO-1 gene expressions were augmented after PBMT as a compensatory mechanism that prevented a pulmonary tissue disease from the oxidative damage induced by FA. It is known that HO-1 is a rate-limiting enzyme in heme catabolism, degrading heme to free iron, biliverdin, and carbon monoxide. SOD in turn promotes the degradation of the superoxide anion in hydrogen peroxide and it is less reactive. These products exert a cytoprotective mechanism against oxidative stress [31]. On the other hand, no differences were observed in CAT levels.

Another important antioxidant source in the lung is glutathione, which is essential for a defensive response to oxidants and inflammatory agents, by repairing the oxidized and damaged molecules and helping to regulate a variety of cellular functions [32]. Our study has found that the activities of GST and GPX were increased by PBMT. As previously mentioned, these data can explain the reduced levels of H_2_O_2_, since GPX is a potent antioxidant against H_2_O_2_.

Here we have utilized an interesting model of lung disease induced by pollution. Moreover, several lung diseases, such as asthma, a chronic obstructive pulmonary disease, and lung fibrosis, have all been associated with oxidative stress [18, 19, 33] and in addition the pollution might aggravate these diseases. In this context, taking everything into account, our data has shown that PBMT might be as a promissory treatment for lung diseases mediated by oxidative stress. Additionally, this treatment is without side effects, it presents low costs, and it demonstrates a noninvasive therapy.
